# Massively Parallel Sequencing and Analysis of the *Necator americanus* Transcriptome

**DOI:** 10.1371/journal.pntd.0000684

**Published:** 2010-05-11

**Authors:** Cinzia Cantacessi, Makedonka Mitreva, Aaron R. Jex, Neil D. Young, Bronwyn E. Campbell, Ross S. Hall, Maria A. Doyle, Stuart A. Ralph, Elida M. Rabelo, Shoba Ranganathan, Paul W. Sternberg, Alex Loukas, Robin B. Gasser

**Affiliations:** 1 Department of Veterinary Science, The University of Melbourne, Werribee, Victoria, Australia; 2 Department of Genetics, Genome Sequencing Center, Washington University School of Medicine, St. Louis, Missouri, United States of America; 3 Department of Biochemistry and Molecular Biology, Bio21 Molecular Science and Biotechnology Institute, The University of Melbourne, Victoria, Australia; 4 Departamento de Parasitologia, Instituto de Ciências Biológicas, Universidade Federal de Minas Gerais, Belo Horizonte, Minas Gerais, Brazil; 5 Department of Chemistry and Biomolecular Sciences, Macquarie University, Sydney, New South Wales, Australia; 6 Howard Hughes Medical Institute and Division of Biology, California Institute of Technology, Pasadena, California, United States of America; 7 James Cook University, Cairns, Queensland, Australia; Biomedical Research Institute, United States of America

## Abstract

**Background:**

The blood-feeding hookworm *Necator americanus* infects hundreds of millions of people worldwide. In order to elucidate fundamental molecular biological aspects of this hookworm, the transcriptome of the adult stage of *Necator americanus* was explored using next-generation sequencing and bioinformatic analyses.

**Methodology/Principal Findings:**

A total of 19,997 contigs were assembled from the sequence data; 6,771 of these contigs had known orthologues in the free-living nematode *Caenorhabditis elegans*, and most of them encoded proteins with WD40 repeats (10.6%), proteinase inhibitors (7.8%) or calcium-binding EF-hand proteins (6.7%). Bioinformatic analyses inferred that the *C. elegans* homologues are involved mainly in biological pathways linked to ribosome biogenesis (70%), oxidative phosphorylation (63%) and/or proteases (60%); most of these molecules were predicted to be involved in more than one biological pathway. Comparative analyses of the transcriptomes of *N. americanus* and the canine hookworm, *Ancylostoma caninum*, revealed qualitative and quantitative differences. For instance, proteinase inhibitors were inferred to be highly represented in the former species, whereas SCP/Tpx-1/Ag5/PR-1/Sc7 proteins ( = SCP/TAPS or *Ancylostoma*-secreted proteins) were predominant in the latter. In *N. americanus*, essential molecules were predicted using a combination of orthology mapping and functional data available for *C. elegans*. Further analyses allowed the prioritization of 18 predicted drug targets which did not have homologues in the human host. These candidate targets were inferred to be linked to mitochondrial (e.g., processing proteins) or amino acid metabolism (e.g., asparagine t-RNA synthetase).

**Conclusions:**

This study has provided detailed insights into the transcriptome of the adult stage of *N. americanus* and examines similarities and differences between this species and *A. caninum*. Future efforts should focus on comparative transcriptomic and proteomic investigations of the other predominant human hookworm, *A. duodenale*, for both fundamental and applied purposes, including the prevalidation of anti-hookworm drug targets.

## Author Summary

The blood-feeding hookworm *Necator americanus* infects hundreds of millions of people. To elucidate fundamental molecular biological aspects of this hookworm, the transcriptome of adult *Necator americanus* was studied using next-generation sequencing and *in silico* analyses. Contigs (n = 19,997) were assembled from the sequence data; 6,771 of them had known orthologues in the free-living nematode *Caenorhabditis elegans*, and most encoded proteins with WD40 repeats (10.6%), proteinase inhibitors (7.8%) or calcium-binding EF-hand proteins (6.7%). Bioinformatic analyses inferred that *C. elegans* homologues are involved mainly in biological pathways linked to ribosome biogenesis (70%), oxidative phosphorylation (63%) and/or proteases (60%). Comparative analyses of the transcriptomes of *N. americanus* and the canine hookworm, *Ancylostoma caninum*, revealed qualitative and quantitative differences. Essential molecules were predicted using a combination of orthology mapping and functional data available for *C. elegans*. Further analyses allowed the prioritization of 18 predicted drug targets which did not have human homologues. These candidate targets were inferred to be linked to mitochondrial metabolism or amino acid synthesis. This investigation provides detailed insights into the transcriptome of the adult stage of *N. americanus*.

## Introduction

Soil-transmitted helminths ( = geohelminths) are responsible for neglected tropical diseases (NTDs) mostly in developing countries [1]. In particular, the blood-feeding hookworms *Necator americanus* and *Ancylostoma duodenale* (Nematoda) infect ∼740 million people in rural areas of the tropics and subtropics [2], causing an estimated disease burden of 22 million disability-adjusted life years (DALYs) [3]. Geographically, *N. americanus* is the most widely distributed hookworm of humans globally [4]. The life cycle is direct, with thin-shelled eggs passed in the faeces from the infected host. Under suitable environmental conditions (e.g., 26°C and 100% humidity; [5]), the eggs hatch and develop through two free-living larval stages to the infective, third-stage (L3; filariform) larvae. The latter larvae penetrate human skin and migrate *via* the circulatory system and lung to finally reside as adults usually in the duodenum. The adult stages attach by their buccal capsule to the intestinal mucosa, rupture capillaries and feed on blood. The pathogenesis of hookworm disease is mainly a consequence of the blood loss, which occurs during attachment and feeding. The disease ( = necatoriasis) is commonly characterized by iron-deficiency anaemia, which can cause physical and mental retardation and sometimes deaths in children, adverse maternal-foetal outcomes [6]–[7] and, in chronically infected individuals, can result in a significant alteration of their immune response to helminths [8].

Traditionally, the control of hookworm disease has relied mostly on the treatment of infected individuals with anthelmintics, such as albendazole, mebendazole, pyrantel pamoate and/or levamisole. With mass treatment strategies now in place in a number of countries [9]–[10], there is an increased potential for hookworms to develop genetic resistance against the compounds administered, if they are used excessively and at suboptimal dosages. Thus, given the experience with drug resistance in parasitic nematodes of livestock [11], it is prudent to maintain a continual focus on the discovery of novel drugs against hookworms of humans. Such a discovery effort could be underpinned by an integrated genomic-bioinformatic approach, using functional genomic and phenomic information available for the free-living nematode *Caenorhabditis elegans* (see WormBase; www.wormbase.org). This nematode, which is the best characterized metazoan organism [12]–[13], is considered to be relatively closely related to nematodes of the order Strongylida (to which hookworms belong) [14]. Current evidence indicates that ∼60% of genes in strongylids (or hookworms) have orthologues/homologues in *C. elegans* [15]–[16], and that a range of biological pathways is conserved between strongylid nematodes/hookworms and this free-living nematode [17]–[20]. Therefore, conducting comparative explorations of molecular data sets between these nematodes should identify nematode-specific biological pathways, which, if essential for the development and survival, could provide new targets for nematocidal drugs.

Next generation sequencing technologies, such as ABI-SOLiD, Illumina/Solexa (www.illumina.com; [21]), Helicos (www.helicosbio.com; [22]) and 454/Roche (www.454.com; [23]), together with the recent progress in bioinformatics, are providing unique opportunities for the high-throughput transcriptomic and genomic explorations of nematodes in far more detail than previously possible [24] and at a substantially lower cost than using conventional (Sanger) sequencing. To date, genomic and molecular studies of hookworms have mainly involved the canine hookworm, *Ancylostoma caninum* [19], [25]–[27], because of its use as a model for human hookworms [27]–[28]. In contrast, genomic datasets for *N. americanus* are scant, representing a major constraint to progress in molecular research of this nematode [4]. In the present study, we (i) conducted a detailed exploration and functional annotation of the transcriptome of the adult stage of *N. americanus* by 454 sequencing coupled to semi-automated bioinformatic analyses, (ii) compared the transcriptome of *N. americanus* to currently available transcriptomic data for *A. caninum*, and (iii) inferred the essentiality of key genes and gene products in order to predict putative drug targets.

## Materials and Methods

### Accession numbers

The nucleotide sequence data produced for this study are available in the GenBank database under accession SRA012052. The contigs assembled from these data can be requested from the primary author or are available at www.nematode.net.

### Parasite material

The “Shanghai strain” of *N. americanus* (kindly provided by Drs Bin Zhan and Peter Hotez) was produced in golden hamsters (*Mesocricetus auratus*; infected for 94 days) at the Universidade Federal de Minas Gerais, Brazil. The infection experiment was conducted according the animal ethics guidelines of the Universidade Federal de Minas Gerais.

### RNA isolation, cDNA synthesis and 454 sequencing

Total RNA from 30 adult worms was prepared using TRIzol Reagent (GibcoBRL, Life Technologies, USA) following the manufacturer's instructions and then treated with Ambion Turbo DNase (Ambion/Applied Biosystems, Austin, TX). The integrity of the RNA was verified using the Bioanalyzer 2100 (Agilent Technologies, USA), and the yield determined using the NanoDrop ND-1000 UV-VIS spectrophotometer v.3.2.1 (NanoDrop Technologies, Wilmington, DE). The cDNA library was constructed using the SMART™ kit (Clontech/Takara Bio, CA) from ∼100ng of total RNA. An optimized PCR cycling protocol (over 20 cycles) was used to amplify full-length cDNAs, employing primers complementary to the SMART IIA-Probe and custom oligo(dT), and the Advantage-HF 2 polymerase mix (Clontech/Takara). The cDNA was normalized by denaturation-reassociation, treated with duplex-specific nuclease (Trimmer kit, Evrogen, CA) and amplified over 11 cycles. Subsequently, the 5′- and 3′- adaptors were removed by digestion with the exonuclease *Mme*1 and streptavidin-coated paramagnetic beads [29]. The normalized cDNA (500–700 bases) was then amplified using 9 cycles of Long and Accurate (LA)-PCR [30] and then sequenced in a Genome Sequencer™ (GS) Titanium FLX instrument (Roche Diagnostics) employing a standard protocol [23].

### Bioinformatic analyses

Expressed sequence tags (ESTs) generated from the normalised cDNA library for *N. americanus* were assembled and annotated using a standard bioinformatic pipeline [31]. Briefly, sequences were aligned and assembled using the Contig Assembly Program v.3 (CAP3; [32], employing a minimum sequence overlap length of 50 nucleotides and an identity threshold of 95%. ESTs (n = 2,200; www.ncbi.nlm.nih.gov) from adult *N. americanus* available from previous studies [4], [16], [33], [34] were included for comparative analysis. Following the pre-processing of the ESTs, contigs and singletons from the present dataset were subjected to analysis by BLASTx (NCBI, www.ncbi.nlm.nih.gov) and BLASTn (EMBL-EBI Parasite Genome Blast Server, www.ebi.ac.uk) to identify putative homologues in *C. elegans*, other nematodes, and organisms other than nematodes (e-value of ≤1e-05). WormBase Release WS200 (www.wormbase.org) was interrogated extensively for relevant information on *C. elegans* orthologues/homologues, including transcriptomic, proteomic, RNAi phenotypic and interactomic data. Gene ontology (GO) annotations were conducted using BLAST2GO [35]. Peptides were mapped by InterProScan [36] and linked to respective pathways in *C. elegans* using the KEGG Orthology-Based Annotation System (KOBAS, [37]). The protein sequences inferred from open reading frames (ORFs) of the ESTs with orthologues in *C. elegans* were also subjected to “secretome analysis” using the program SignalP v.2.0 (available at www.cbs.dtu.dk/services/SignalP/), employing both the neural network and hidden Markov models to predict signal peptides and/or anchors [38]–[40]. Also, transmembrane domains were inferred using the program TMHMM (www.cbs.dtu.dk/services/TMHMM/; [41]–[43]). Protein sequences inferred from contigs for *N. americanus* were compared with those predicted for *C. elegans* and from a similar-sized, publicly available EST dataset for adult *A. caninum* produced by 454 sequencing (GenBank accession numbers EW741128-EW744730; EX534506-EX567272); protein similarities were displayed using SimiTri [44].

### Prediction of essentiality and drug targets

All protein sequences predicted from contigs for *N. americanus* were compared with protein sequences available in the OrthoMCL 2.0 database (www.OrthoMCL.org) by BLASTp (e-value cut off of <1e-05). A subset of *C. elegans* protein homologues was then selected based on: (i) an association with a lethal RNAi phenotype; (ii) the presence/absence of gene paralogues (based on OrthoMCL orthology grouping); and (iii) GO annotation to terms linked to enzyme or G protein-coupled receptor (GPCR) activity (i.e., GO:0003824 or GO:0004930, or a sub-term thereof). The following information was obtained: (i) network connectivity score (cf. http://www.functionalnet.org/wormnet/Wormnet_v1_index.html; see [45]); (ii) presence of mammalian orthologues (based on OrthoMCL orthology grouping (iii) essentiality information (i.e. association with non-wildtype RNAi phenotypes) in other model organisms (including *Saccharomyces cerevisiae*, *Mus musculus* and *Drosophila melanogaster*) based on OrthoMCL groups. Each predicted drug target was selected based on (i) the presence of orthologues linked to non-wildtype RNAi or mutant phenotypes in *S. cerevisiae*, *M. musculus* and *D. melanogaster*, (ii) the absence of orthologues/homologues from the human host and (iii) its network connectivity score [45].

To predict the potential of selected *C. elegans* orthologues of *N. americanus* contigs as drug targets ( = “druggability”), the InterPro domains inferred from the predicted proteins were compared with those linked to known small molecular drugs which follow the ‘Lipinsky rule of 5’ regarding bioavailability [46], [47]. Similarly, GO terms inferred from the predicted proteins were mapped to Enzyme Commission (EC) numbers, and a list of enzyme-targeting drugs was compiled based on data available in the BRENDA database (www.brenda-enzymes.info; [48], [49]). The *C. elegans* orthologues included in the list were ranked according to the ‘severity’ of the non-wild-type RNAi phenotypes (i.e. adult lethal, embryonic and/or larval lethal, sterile and other defects) in *C. elegans* (cf. www.wormbase.org) defined in previous studies [50], [51].

## Results

A total of 116,839 ESTs (287±235 bases in length) was generated by 454 sequencing. After removing the ESTs of <100 bases, 63,523 ESTs were assembled into 19,997 contigs (369 bases±215.31). Of these, 6,771 (33.9%) had known *C. elegans* orthologues, and 2,287 (11.4%) matched known nucleotide sequences from various nematodes, including *Brugia malayi*, *Haemonchus contortus*, *Pristionchus pacificus*, *N. americanus*, *A. caninum*, *A. duodenale* and *Nippostrongylus braziliensis* (73.2%), other invertebrates (21.3%) and some vertebrates (5.5%) available in current databases. All of the previously published ESTs for *N. americanus* (www.ncbi.nlm.nih.gov; [4], [16], [33], [34]) represented a subset (12.4%) of the present dataset (not shown). The number of ORFs in the *N. americanus* EST data, predicted peptides and their signal, transmembrane and/or InterPro domains as well as the results of GO and KOBAS (pathway mapping) searches are given in Table 1. A total of 12,799 proteins were predicted from the 19,997 contigs, of which 7,214 mapped to known proteins defined by 2,381 different domains (Tables 1 and S1), the most abundant being ‘WD40’ (IPR0011680; 10.6%), ‘proteinase inhibitors’ (IPR000215; 7.8%) and ‘EF-hand’ molecules (IPR018248; 6.7%) (Table 2). The subsequent annotation of the inferred proteins revealed 887 different GO terms, of which 314 were ‘biological process’, 117 ‘cellular component’ and 456 ‘molecular function’ (Tables 3 and S2). The predominant terms were ‘translation’ (GO:0006412, 20.3%) and ‘metabolic process’ (GO:0008152, 14.9%) for ‘biological process’; ‘intracellular’ (GO:0005622, 25.1%) and ‘ribosome’ (GO:0005840, 17%) for ‘cellular component’, and, ‘ATP binding’ (GO:0005524, 18.9%) and ‘structural constituent of ribosome’ (GO:0003735, 17.9%) for ‘molecular function’ (Tables 3 and S2). Proteins inferred from the *N. americanus* contigs were predicted to be involved in 235 different biological pathways, of which the vast majority represented ‘ribosome biogenesis’ (n = 163, 70%), ‘oxidative phosphorylation’ (n = 148, 63%) and ‘proteases’ (n = 140, 60%) (see Table S3).

**Table 1:** Summary of the expressed sequence tag (EST) data for the adult stage of *Necator americanus* determined following 454 sequencing and detailed bioinformatics annotation and analyses.

No. of EST clusters	19,997
Average length (±standard deviation)	369 bp±215.31
Containing an Open Reading Frame	12,799
Signal peptides	274
Returning InterProScan results	7,214 (2,381 domains)
Gene Ontology	2,950 (887 terms)
*Biological process*	4,830 (314 terms)
*Cellular component*	3,087 (117 terms)
*Molecular function*	8,671 (456 terms)
Prediction of biological pathways (KOBAS)	235

**Table 2:** The thirty most abundant protein domains inferred using the InterProScan software from peptides inferred for *Necator americanus* and *Ancylostoma caninum*.

InterProScan domain	No. of *Na* EST clusters (%)	No. of *Ac* EST clusters (%)
WD40	315 (10.6) ▾	553 (14.5) ▴
EF-HAND	196 (6.7)	187 (2.6)
Proteinase inhibitors	230 (7.8) ▴	126 (3.3) ▾
Proteases	179 (6.1)	177 (4.6)
Protein kinases	131 (4.4) ▾	388 (10.1) ▴
NAD(P)-binding domain	114 (3.9) ▾	160 (4.2) ▴
Transthyretin-like	97 (3.3) ▴	19 (0.5) ▾
Galectin, carbohydrate recognition domain	95 (3.2) ▴	66 (1.7) ▾
SCP-like extracellular	94 (3.2) ▾	362 (9.5) ▴
Peptidyl-prolyl cis-trans isomerase	91 (3.1) ▴	25 (0.6) ▾
RNA recognition motif, RNP-1	83 (2.8) ▾	198 (6.2) ▴
Mitochondrial substrate/solute carrier	88 (3)	88 (2.3)
Thioredoxin fold	81 (2.7)	70 (1.8)
Allergen V5/Tpx-1 related	64 (2.2) ▾	232 (6) ▴
Zinc finger, C2H2-type	64 (2.2) ▾	185 (4.8) ▴
Aldo/keto reductase	60 (2) ▴	9 (0.2) ▾
Scr homology-3 domain	57 (2)	98 (2.6)
Actin/actin like	56 (2)	49 (1.3)
Short-chain dehydrogenase/reductase SDR	51 (1.7)	51 (6.2)
Metridin-like ShK toxin	47 (1.6) ▴	6 (0.1) ▾
Histone-fold	44 (1.5) ▴	19 (0.5) ▾
Nucleotide binding, alpha beta plait	43 (1.4) ▾	80 (2.1) ▴
Heat shock protein Hsp20	41 (1.4) ▴	14 (0.4) ▾
Chaperonin Cpn60/TCP-1	39 (1.3)	50 (1.3)
Cytochrome P450	39 (1.3) ▴	4 (0.1) ▾
Ankyrin	38 (1.2) ▾	271 (7) ▴
Annexin repeat	37 (1.2)	53 (1.4)
Ubiquitin-conjugating enzyme, E2	37 (1.2) ▴	15 (0.4) ▾
Tetratricopeptide repeat	36 (1.2)	20 (0.5)
Protein-tyrosine phosphatase, receptor/non-receptor type	35 (1.2) ▾	76 (2) ▴

The arrows infer statistically significant (*p*<0.05; chi-square) higher (▴) or lower (▾) number of genes encoding proteins (with particular InterPro domains) common to *N. americanus* and *A. caninum*.

**Table 3:** The twenty most abundant Gene Ontology (GO) terms (according to the categories ‘biological process’, ‘cellular component’ and ‘molecular function’) for peptides inferred for *Necator americanus* and *Ancylostoma caninum*.

GO term	GO code	No. of *Na* EST clusters (%)	No. of *Ac* EST clusters (%)
*Biological process*			
Translation	GO:0006412	599 (20.3)	146 (3.8)
Metabolic process	GO:0008152	438 (14.9)	284 (7.4)
Proteolysis	GO:0006508	329 (11.2)	254 (6.6)
Oxidation reduction	GO:0055114	197 (6.7)	85 (2.2)
Protein amino acid phosphorylation	GO:0006468	147 (5)	159 (4.2)
Regulation of transcription, DNA-dependent	GO:0006355	137 (4.6)	53 (1.4)
Transport	GO:0006810	134 (4.5)	114 (3)
ATP synthesis coupled proton transport		111 (3.7)	34 (0.9)
Protein folding	GO:0006457	104 (3.5)	48 (1.3)
Carbohydrate metabolic process	GO:0005975	101 (3.4)	100 (2.6)
Small GTPase mediated signal transduction	GO:0007264	62 (2.1)	38 (1)
Ubiquitin-dependent protein catabolic process	GO:0006511	62 (2.1)	30 (0.8)
Intracellular protein transport	GO:0006886	59 (2)	52 (1.4)
Vesicle-mediated transport	GO:0016192	54 (1.8)	39 (1)
Nucleosome assembly	GO:0006334	53 (1.8)	21 (0.5)
Protein transport	GO:0015031	50 (1.7)	40 (1)
Response to oxidative stress	GO:0006979	48 (1.6)	9 (0.2)
Protein amino acid dephosphorylation	GO:0006470	47 (1.6)	45 (1.2)
Protein polymerization	GO:0051258	46 (1.6)	15 (0.4)
*Cellular component*			
Intracellular	GO:0005622	798 (25.1)	297 (7.5)
Ribosome	GO:0005840	499 (17)	88 (2.3)
Membrane	GO:0016020	296 (9.7)	251 (6.6)
Nucleus	GO:0005634	280 (9.5)	174 (4.6)
Integral to membrane	GO:0016021	185 (6.3)	143 (3.7)
Cytoplasm	GO:0005737	141 (4.8)	122 (3.2)
Extracellular region	GO:0005576	86 (2.9)	156 (4)
Nucleosome	GO:0000786	51 (1.7)	18 (0.5)
Protein complex	GO:0043234	46 (1.6)	15 (0.4)
Endoplasmic reticulum	GO:0005783	38 (1.3)	35 (0.9)
Mitochondrion	GO:0005739	36 (1.2)	5 (0.1)
Cytoskeleton	GO:0005856	33 (1.1)	14 (0.4)
Microtubule	GO:0005874	31 (1)	15 (0.4)
Proton-transporting two-sector ATPase complex, catalytic domain	GO:0033178	29 (1)	19 (0.5)
Proton-transporting two-sector ATPase complex, proton-transporting domain	GO:0033177	27 (0.9)	8 (0.2)
Mitochondrial inner membrane	GO:0005743	22 (0.7)	7 (0.2)
Proton-transporting ATP synthase complex, catalytic core F(1)	GO:0045261	18 (0.6)	9 (0.2)
Clathrin adaptor complex	GO:0030131	15 (0.5)	9 (0.2)
Proteasome core complex	GO:0005839	15 (0.5)	15 (0.4)
Eukaryotic translation elongation factor 1 complex	GO:0005853	15 (0.5)	8 (0.2)
*Molecular function*			
ATP binding	GO:0005524	558 (18.9)	514 (13.4)
Structural constituent of ribosome	GO:0003735	527 (17.9)	91 (2.4)
Catalytic activity	GO:0003824	429 (14.5)	346 (9)
Oxidoreductase activity	GO:0016491	317 (10.8)	185 (4.8)
Protein binding	GO:0005515	311 (10.5)	212 (5.6)
Binding	GO:0005488	287 (9.3)	237 (6.2)
Zinc ion binding	GO:0008270	287 (9.3)	214 (5.6)
DNA binding	GO:0003677	242 (8.2)	121 (3.2)
Serine-type endopeptidase inhibitor activity	GO:0004252	230 (7.8)	25 (0.7)
Nucleic acid binding	GO:0003676	204 (6.9)	192 (5)
GTP binding	GO:0005525	202 (6.8)	95 (2.5)
Calcium ion binding	GO:0005509	169 (5.8)	79 (2)
Electron carrier activity	GO:0009055	140 (4.7)	59 (1.5)
Heme binding	GO:0020037	134 (4.5)	17 (0.4)
RNA binding	GO:0003723	124 (4.2)	90 (2.3)
Iron ion binding	GO:0008270	123 (4.2)	18 (0.5)
Nucleotide binding	GO:0000166	113 (3.8)	138 (3.6)
Aspartic-type endopeptidase activity	GO:0004190	101 (3.4)	27 (0.7)
Sugar binding	GO:0005529	99 (3.4)	18 (0.5)
Transcription factor activity	GO:0003700	98 (3.3)	47 (1.2)

**Figure 1:**
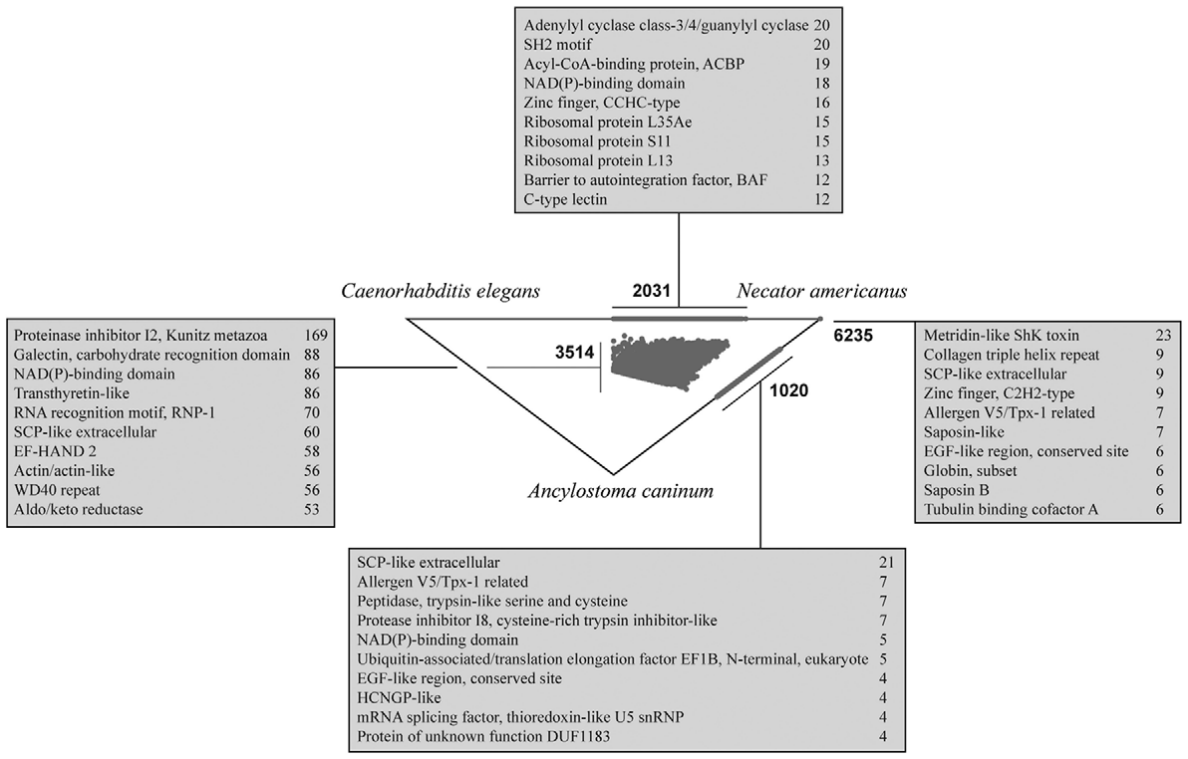
Simitri analysis. Relationships of proteins predicted for *Necator americanus* with homologues from *Ancylostoma caninum* and *Caenorhabditis elegans*, displayed in a SimiTri plot [44]. The description of proteins with most abundant InterPro domains identified in each similarity group is given in the boxes.

For comparative analyses, publicly available EST data for the adult stage of *A. caninum* was included. For this dataset, the same bioinformatic analyses described in the Methods section were conducted. From 15,755 contigs of *A. caninum*, a total of 12,622 proteins were inferred, of which 4,534 matched those encoded by *N. americanus* ORFs (Figure 1); 8,650 of these predicted proteins could be mapped to known molecules with 2,546 different motifs (Tables 1 and S1). The protein motifs ‘SCP-like extracellular’ (IPR014044, 9.5%), ‘ankyrin’ (IPR002110, 7%) and ‘allergen V5/Tpx-1 related’ (IPR0011283, 6%) were most commonly recorded in the *A. caninum* dataset (Table 2). Differences in the numbers of IPR domains identified in the *N. americanus* and *A. caninum* predicted peptides were calculated using a Chi-square test (*p*<0.05) and are indicated in Table 2. GO annotation of the *A. caninum* predicted peptides revealed 323 different terms for ‘biological process’, 119 for ‘cellular component’ and 500 for ‘molecular function’ (Tables 3 and S2). The terms ‘metabolic process’ (GO:0008152, 7.4%) and ‘proteolysis’ (GO:0006508, 6.6%) had the highest representation for ‘biological process’, as did ‘intracellular’ (GO:0005622, 7.5%) and ‘membrane’ (GO:0016020, 6.6%) for ‘cellular component’; and, ‘ATP binding’ (GO:0005524, 13.4%) and ‘catalytic activity’ (GO:0003824, 9%) for ‘molecular function’ (Tables 3 and S2). Using the protein data, a total of 235 different biological pathways were predicted, of which ‘proteases’ (n = 219, 93%), ‘other enzymes’ (n = 164, 70%) and ‘protein kinases’ (n = 151, 54%) were the most predominant (see Table S3).

From the *N. americanus* dataset, 5,498 proteins matched known proteins encoded by orthologues available in the OrthoMCL 2.0 database (www.OrthoMCL.org); 372 of these proteins had homologues in *C. elegans*, and 278 (277 enzymes and one G-PCR) of them were linked to adult lethal, embryonic and/or larval lethal and sterile RNAi phenotypes (Table S4). A subset of 18 molecules in *N. americanus* with homologues in *C. elegans* but not in humans were defined, also considering RNAi phenotype/s [i.e. adult lethal (n = 2), larval and/or embryonic lethal (n = 16), sterile (n = 4) and other defects (n = 12); cf. Table 4], as drug target candidates. These proteins could be mapped to 54 ‘druggable’ InterPro domains, and 212 EC numbers were linked to ‘druggable’ enzymes; a total of 3,320 effective drugs were predicted (Table S4).

**Table 4:** Description of *Caenorhabditis elegans* orthologues of *Necator americanus* contigs for which inferred peptides were associated with ‘druggable’ InterPro domains and/or Enzyme Commission (EC) numbers, and examples of candidate nematocidal compounds linked to these domains predicted using the BRENDA database (see Materials and Methods).

C. elegans *gene ID*	*Gene name*	*RNAi phenotypes*	*Protein description*	A. caninum *orthologue*	*Druggable IPR domain (description)*	*Examples of compounds in the BRENDA database*
WBGene00003816	*nrs-2*	Lethal, larval lethal, larval arrest, sterile	Asparagine synthetase	√		1-methyl-4-(1-methylethyl)-7-oxabicyclo[2.2.1]heptane
WBGene00012166	W01A8.4	Lethal, embryonic lethal, larval arrest, sterile	Mitochondrial NADH dehydrogenase (ubiquinone) complex (complex I) subunit	√		1-Geranyl-2-methylbenzimidazole
WBGene00021952	*vha-19*	Larval lethal, maternal sterile, sick	V0 subunit of V-ATPase	√		Efrapeptin
WBGen00007001	*tufm-2*	Embryonic lethal, slow growth, sick, sterile progeny	Elongation factor Tu	√		Leu-Gly-Asn repeat-enriched protein
WBGene00010562	*cdc-48.3*	Embryonic lethal, larval lethal, larval arrest, slow growth, sick	AAA ATPase	√		1-beta-D-ribofuranosyl-1,2,4-triazole-3-carboxamide-5′-triphosphate
WBGene00018963	*ucr-1*	Embryonic lethal, larval arrest, maternal sterile	Mitochondrial processing protease enhancing protein	√	IPR001431 (Insulinase family)	
WBGene00001566	*acdh-13*	Embryonic lethal, sterile progeny, slow growth	Acyl-CoA dehydrogenase	√		
WBGene00004020	*pho-1*	Embryonic lethal, larval arrest, sterile	Intestinal acid phosphatase	√		1,10-phenanthroline
WBGene00009161	F26E4.6	Embryonic lethal, larval arrest, slow growth, maternal sterile	cytochrome c oxidase	√		amyloid beta
WBGene00022169	Y71H2aM.4	Embryonic lethal, larval arrest, slow growth	NADH:ubiquinone oxidoreductase, NDUFC2/B14.5B subunit	√		1-Geranyl-2-methylbenzimidazole
WBGene00000151	*apn-1*	Embryonic lethal	AP endonuclease (family 2)	√		
WBGene00019885	R05D8.7	Embryonic lethal	Reductases with broad range of substrate specificities	√	IPR002198 (Short-chain dehydrogenase/reductase)	4-Trifluoromethyl-2,3-dihydro-2,3-dihydroxybenzoate
WBGene00020089	R119.3	Embryonic lethal	Dehydrogenase		IPR002198 (Short-chain dehydrogenase/reductase)	4-Trifluoromethyl-2,3-dihydro-2,3-dihydroxybenzoate
WBGene00011803	T16G12.1	Embryonic lethal	Aminopeptidase	√		
WBGene00020149	T01D1.4	Embryonic lethal	Uncharacterized conserved protein, contains double-stranded beta-helix domain	√		
WBGene00006592	*dpy-31*	Embryonic lethal	Zinc metalloprotease	√		
WBGene00019001	F57B10.3	Embryonic lethal, larval lethal, slow growth	Phosphoglycerate mutase	√		1,10-phenanthroline
WBGene00016356	C33F10.8	Embryonic lethal, slow growth	Protein tyrosine phosphatase	√	IPR000387 (Tyrosine protein phosphatases)	1,3-difluoro-2-((E)-2-nitrovinyl)benzene

These genes are not present in *H. sapiens*. The presence of known *Ancylostoma caninum* orthologues is also indicated (‘√’).

## Discussion

Next-generation sequencing and integrated bioinformatic analyses have provided detailed and biologically relevant insights into the transcriptome of the adult stage of *N. americanus*. A total of 12,799 ORFs were inferred from the present EST dataset, thus increasing the number of predicted proteins currently available (for this stage/species) in public databases by approximately 27-fold [4]. Amongst the InterPro domains identified, ‘WD40’, ‘proteinase inhibitors’ and ‘EF-hand’ motifs were the most abundant, followed by ‘proteases’ and ‘protein kinases’. WD40 repeats (also known as WD or beta-transducin repeats) are short (∼40 amino acid) motifs found in the proteomes of all eukaryotes and implicated in a variety of functions, ranging from signal transduction and transcription regulation to cell-cycle control and apoptosis [52], [53]. WD40 motifs act as sites for protein-protein interactions; proteins containing WD40 repeats are known to serve as platforms for the assembly of protein complexes or mediators of a transient interplay with other proteins, such as the ubiquitin ligases, involved in the onset of the anaphase during cell mitosis [54]. Similarly, proteins containing ‘EF-hand’ domains are involved in a number of protein-protein interactions regulated by various specialized systems (e.g., Golgi system, voltage-dependent calcium channels and calcium transporters) for the uptake and release of calcium, which acts as a secondary messenger for their activation [55]. In *C. elegans*, both EF-hand and WD40 proteins are known to be required for the maturation of the nervous system and the formation of ciliated sensory neurons, in particular of the chemoreceptors located in the amphids [8], [56]. The amphids of parasitic nematodes are, besides having the chemoreceptive activity, also known to play a role as secretory organs, primarily to provide an appropriate substrate for the transmission of neuronal potentials [57]. However, in *N. americanus*, a group of specialized amphidial neuronal cells ( = amphidial glands; [57]) expresses a group of aspartic proteases (i.e. cathepsin D-like *Na*-APR-1 and *Na*-APR-2) which are proposed to degrade host haemoglobin and serum proteins in the buccal capsule of adult worms [58]. In the dog hookworm, *A. caninum*, the amphidial glands have also been shown to produce a proteinase inhibitor (called ‘ancylostomatin’) that acts as an anticoagulant to promote the flow of host blood and tissue fluids into the buccal capsule and the intestine of the parasite [59]. Although proteinase inhibitors, such as the ‘kunitz-type’ molecules, were significantly more abundant in the transcriptome of adult *N. americanus* [4], [33] than *Ancylostoma* spp., they have been better characterized in the latter parasites [60]–[63] for which both single and multiple kunitz-domain proteins have been described [61]. For instance, a cDNA coding a single kunitz-domain proteinase inhibitor (named *Ace*KI-1) was isolated from *A. ceylanicum*. The corresponding recombinant protein has been shown to act as a tight-binding inhibitor of the serine proteases chymotrypsin, pancreatic elastase, neutrophil elastase and trypsin [60] and confers partial protection against hookworm-associated growth delay in hamsters [62]. Recently, a kunitz-type cDNA was shown to be enriched in the adult male of *A. braziliense* [63]. Although their precise biological function remains to be determined, kunitz-type proteinase inhibitors of hookworms appear to play pivotal roles in preventing homeostasis and inhibiting host proteases (e.g., pancreatic and intestinal enzymes; [60], [64]).

Proteases were also highly represented in the transcriptome of *N. americanus* (6.1%) as well as that of *A. caninum* (4.6%) (see Table 2). These proteases included cysteine, aspartic and metallo- proteases, which are known to function in multi-enzyme cascades to digest haemoglobin and other serum proteins [65], [66]. In *N. americanus*, cysteine proteases with high sequence homology to the protein cathepsin B were localized to the gut of adult worms and the corresponding mRNAs shown to be upregulated in the adult stage compared with the infective L3 stage, thus strongly suggesting that these enzymes are involved in blood-feeding [67]. In *A. caninum*, a cysteine protease (*Ac*-CP-1) with 86% amino acid sequence identity to those characterized in *N. americanus*, was shown to be expressed in the cephalic and excretory glands [68] and was detected in the excretory/secretory products (ES) [69] of adult worms; thus, it has been proposed that *Ac*-CP-1 functions as an extracorporeal digestive enzyme at the site of attachment [67]. Another cysteine protease (*Ac*-CP-2) was localized to the brush border membrane of the intestine and demonstrated to be involved in the digestion of haemoglobin [65]. The *N. americanus* homologue of *Ac*-CP-2 (i.e. *Na*-CP-2) digests haemoglobin [66] and, expressed as a recombinant protein in *Escherichia coli* and injected subcutaneously into experimental hamsters, has been shown to induce a significant reduction in adult worm burden following challenge infection with L3s of *N. americanus* [28], suggesting that the immunogenic response directed against this protein severely impairs the digestion of host proteins by the adult worms. However, recently, a cathepsin-like cysteine protease has been isolated and characterized in the human filarial nematode *Brugia malayi* and shown by double-stranded RNAi to play an essential role in the early development and maturation of embryos of this nematode [70]. Therefore, it is possible that the abundant transcripts encoding proteases in both adult *N. americanus* and *A. caninum* also reflect a key role of these enzymes in embryogenesis. Proteases have also been isolated from larval stages of both *A. caninum* and *N. americanus* [71], [72]. For instance, a metalloprotease in ES of the activated third-stage larvae (L3) of *A. caninum* has been characterized and demonstrated to be released specifically in response to stimuli that induce feeding [73]. The corresponding cDNA, isolated from an L3 expression library, encoded a zinc-metalloprotease (*Ac*-MTP-1) of the astacin family, that has been proposed to (i) regulate developmental changes associated with the transition from the free-living to the parasitic L3 and the subsequent moult to the fourth-stage larva (L4) [72]; (ii) activate host TGF-ß during the infection, which, in turn, could stimulate parasite development directly, determine tissue predilection sites [74] and/or inhibit neutrophil infiltration at the site of penetration [75]; and, (iii) facilitate skin penetration or tissue migration by the invading L3 [72], [76] and/or degrade the cuticular proteins of the sheath surrounding the infective, free-living L3 [77]. In *N. americanus*, serine proteases have been isolated from ES of the L3 stage and proposed to play a central role in the evasion of the host immune response [71]. Interestingly, a significant number (n = 135, 30%) of *N. americanus* proteases and protease inhibitors of *N. americanus* were not predicted to possess signal peptides indicative of secretion (cf. Tables 1 and 2). The likely explanation for this result is technical and would appear to relate to a 3′-bias in sequence reads [78], thus affecting the prediction of ORFs as well as the identification of signal peptide sequences at the 5′-ends.

Other groups of molecules, such as *Ancylostoma*-secreted proteins (ASPs), have been proposed to have an immunomodulatory function during the invasion of the host, the migration through tissues, attachment to the intestinal wall and blood-feeding [79]. In the present study, ASPs were amongst the ten most abundant groups of molecules in the *N. americanus* dataset, and are most abundant in *A. caninum* (cf. Table 2). ASPs belong to a large group of proteins, the ‘sperm-coating protein (SCP)-like extracellular proteins’, also called SCP/Tpx-1/Ag5/PR-1/Sc7 (SCP/TAPS; Pfam accession number no. PF00188), characterized by the presence of a single or double ‘SCP-like extracellular domain’ (InterPro: IPR014044). In *A. caninum*, double and a single SCP-domain ASPs, called *Ac*-ASP-1 and *Ac*-ASP-2, respectively, were identified as major components of ES from serum-activated, infective L3s and proposed to be secreted in response to one or more host-specific signals during the infection process [80], [81], as also hypothesized in a transcriptomic analysis of serum-activated L3s [19]. In *N. americanus*, homologues of *Ac*-ASP-1 and *Ac*-ASP-2 (i.e *Na*-ASP-1 and *Na*-ASP-2, respectively) have been identified in the L3 stage [82]–[84]. Results from crystallography [85], combined with the observation that *Na*-ASP-2 induces neutrophil and monocyte migration [86], suggest that this molecule has a role as an antagonistic ligand of complement receptor 3 (CR3) and alters the immune cascade by preventing the binding of chemotaxin [85]. Because of its immunogenic properties, *Na*-ASP-2 is under investigation as a vaccine candidate against necatoriasis [7], [28], [81], [87]. In adult *A. caninum*, at least four other ASPs have been identified to date and named *Ac*-ASP-3, *Ac*-ASP-4, *Ac*-ASP-5 and *Ac*-ASP-6 [72]. Another SCP/TAPS molecule, designated neutrophil inhibitor factor (NIF), has been isolated and shown to play an immunomodulatory role by blocking the adhesion of activated neutrophils to vascular endothelial cells and the subsequent release of H_2_O_2_ from activated neutrophils [88] and by interfering with the function of integrin receptors located on the cell surface, which results in the inhibition of platelet aggregation and adhesion [89]. Subsequently, NIF was shown to be transcribed abundantly in the intestines of both *A. caninum* and *N. americanus* [34]. The present study revealed that, although highly represented in the transcriptome of adult *N. americanus*, ASPs were much more abundant in *A. caninum* (cf. Results section). One of the possible explanations for this finding is that, although the *A. caninum* dataset was generated from adult worms recovered from their natural host (i.e. dog), the specimens of *N. americanus* were recovered from a Chinese strain of the golden hamster (*M. auratus*), which is not a natural host for this parasite [90], [91]. Indeed, adults of *N. americanus* recovered from hamsters with patent infections are smaller and less fecund than from the human host [91]. These phenetic differences in this parasite might be associated with variation in transcriptional profiles. However, the difference in prevalence of particular transcripts, such as those of *asp*s, between *A. caninum* and *N. americanus* might reflect their distinct roles in the modulation of the host immune response between the two hookworms, an hypothesis that requires testing.

A benefit of investigating the transcriptome of parasitic nematodes using predictive algorithms is that potential drug targets can be inferred and/or prioritized. The present study identified a subset of 278 ‘druggable’ proteins, of which 18 did not match any human homologues (cf. Results section). Of these 18 molecules, mitochondrial-associated proteins were significantly represented (i.e. encoded by the *C. elegans* orthologues W01a8.4, *ucr-1*, F26E4.6 and Y71H2aM.4; cf. Table 4). Mitochondria are essential organelles with central roles in diverse cellular processes, such as apoptosis, energy production *via* oxidative phosphorylation, ion homeostasis, and the synthesis of haeme, lipid, amino acids, and iron-sulfur ions [92]. In *C. elegans*, defects in the mitochondrial respiratory chain lead to or are associated with a wide variety of abnormalities, including embryonic, larval and adult lethality, sterility and embryonic defects [92]. Despite their essential roles in numerous fundamental biological processes, knowledge of mitochondrial genes and proteins in parasitic nematodes has been utilized mainly to study their systematics, population genetics and ecology [93]–[95]. However, that some mitochondrial-associated proteins are predicted to be essential in *N. americanus* and significantly different from human homologues provides a context for the discovery of new drug targets in mitochondrial pathways and chemical compounds that disrupt these pathways [95], [96]. Amongst the other *N. americanus* orthologues of essential *C. elegans* genes, *nrs-2* encodes an asparaginyl-tRNA synthetase (AsnRS), which is a class II aminoacyl-tRNA synthetase that catalyzes the attachment of asparagine to its cognate tRNA and is required for protein biosynthesis [97]; loss of *nrs-2* function *via* RNAi has been shown to result in a number of phenotypes, including adult and larval lethality and/or larval arrest [97]. In parasitic nematodes, information on amino acid biosynthesis is limited [98]. Although a number of parasitic helminths, including the nematode *Heligmosomoides polygyrus* [*sic. H. bakeri*] and the trematode *Fasciola hepatica*, have been reported to excrete asparagine during *in vitro* incubation [99], [100], the role of asparagine synthetases in essential biological processes is currently unknown. However, in a study investigating the molecular mechanisms of induced cell differentiation in human pro-myelocytic leukemia, asparagine synthetase transcription was reported to be significantly reduced in maturing monocytes/macrophages [101]; therefore, an active role of asparagine synthetases in the development and growth of cancer cells has been suggested, which led to the hypothesis that the induction of a down-regulation of asparagine synthetases might be a new strategy for the treatment of blast cell leukaemia [102]. This finding raises questions about the role(s) of asparagine synthetases in cell differentiation and maturation in parasitic nematodes and the potential of inhibitors of these enzymes as anti-hookworm drugs.

The present study has provided new insights into the transcriptome of *N. americanus*, elucidated similarities and differences between the transcriptomes of *N. americanus* and the related canine hookworm, *A. caninum*, and predicted a panel of novel drug targets and nematocides. All except one of the essential ‘druggable’ proteins (n = 18) inferred for *N. americanus* were present in the *A. caninum* (and *C. elegans*) but not in the mammalian hosts, suggesting relative sequence conservation for these targets among nematodes. The prediction of such targets is particularly important, considering the risk of emerging drug resistance in parasitic nematodes [102], [103]. Clearly, transcriptomic and genomic studies, such as that carried out here can facilitate and expedite the prevalidation of targets for nematocidal drugs, although the lack of genomic and transcriptomic data for many nematodes, including the human hookworm *A. duodenale*, impairs the comparative exploration of essential biological pathways in parasitic nematodes of major public health significance [6]. Furthermore, the present analysis has inferred qualitative and quantitative differences in the transcriptome between *N. americanus* and *A. caninum*, raising questions as to the suitability of the latter species as a model for the former. Although these differences require experimental validation, there is a need to define the transcriptome of *A. duodenale* as a foundation for comparative investigations with a perspective on the identification of new and hookworm-specific drug targets.

## Supporting Information

Table S1 InterPro domains identified in the peptides predicted for *Necator americanus* and *Ancylostoma caninum*.
(0.39 MB XLS)

Table S2 Gene Ontology (GO) terms (according to the categories ‘biological process’, ‘cellular component’ and ‘molecular function’) linked to peptides predicted for *Necator americanus* and *Ancylostoma caninum*.
(0.16 MB XLS)

Table S3 Biological pathways involving key peptides predicted for *Necator americanus* and *Ancylostoma caninum*.
(0.04 MB XLS)

Table S4 Description of *Caenorhabditis elegans* orthologues of *Necator americanus* contigs for which inferred peptides were associated with ‘druggable’ InterPro domains and/or Enzyme Commission (EC) numbers, and a list of candidate nematocidal compounds linked to these domains predicted using the BRENDA database (see Materials and methods). The presence (&#10003) or absence (X) of known orthologues in *Ancylostoma caninum*, *Haemonchus contortus*, *Homo sapiens*, *Drosophila melanogaster* and/or *Mus musculus* is also indicated.
(0.23 MB XLS)
